# Evaluation of the safety and immunogenicity of different COVID-19 vaccine combinations in healthy individuals: study protocol for a randomized, subject-blinded, controlled phase 3 trial [PRIBIVAC]

**DOI:** 10.1186/s13063-022-06345-2

**Published:** 2022-06-16

**Authors:** Xuan Ying Poh, I. Russel Lee, Clarissa Lim, Jefanie Teo, Suma Rao, Po Ying Chia, Sean W. X. Ong, Tau Hong Lee, Ray J. H. Lin, Lisa F. P. Ng, Ee Chee Ren, Raymond T. P. Lin, Lin-Fa Wang, Laurent Renia, David Chien Lye, Barnaby E. Young

**Affiliations:** 1 National Centre for Infectious Diseases, Singapore, Singapore; 2 Tan Tock Seng Hospital, Singapore, Singapore; 3 Lee Kong Chian School of Medicine, Nanyang Technological University, Singapore, Singapore; 4 A*STAR Infectious Diseases Lab, Singapore, Singapore; 5 Yong Loo Lin School of Medicine, National University of Singapore, Singapore, Singapore; 6 Singapore Immunology Network, A*STAR, Singapore, Singapore; 7 National Public Health Laboratory, Singapore, Singapore; 8 Duke-NUS Medical School, Singapore, Singapore

**Keywords:** COVID-19, SARS-CoV-2, Vaccine booster, Immunogenicity, Antibodies, Randomized controlled trial, Phase 3

## Abstract

**Background:**

Over 2021, COVID-19 vaccination programs worldwide focused on raising population immunity through the primary COVID-19 vaccine series. In Singapore, two mRNA vaccines (BNT162b2 and mRNA-1273) and the inactivated vaccine CoronaVac are currently authorized under the National Vaccination Programme for use as the primary vaccination series. More than 90% of the Singapore population has received at least one dose of a COVID-19 vaccine as of December 2021. With the demonstration that vaccine effectiveness wanes in the months after vaccination, and the emergence of Omicron which evades host immunity from prior infection and/or vaccination, attention in many countries has shifted to how best to maintain immunity through booster vaccinations.

**Methods:**

The objectives of this phase 3, randomized, subject-blinded, controlled clinical trial are to assess the safety and immunogenicity of heterologous boost COVID-19 vaccine regimens (intervention groups 1–4) compared with a homologous boost regimen (control arm) in up to 600 adult volunteers. As non-mRNA vaccine candidates may enter the study at different time points depending on vaccine availability and local regulatory approval, participants will be randomized at equal probability to the available intervention arms at the time of randomization. Eligible participants will have received two doses of a homologous mRNA vaccine series with BNT162b2 or mRNA-1273 at least 6 months prior to enrolment. Participants will be excluded if they have a history of confirmed SARS or SARS-CoV-2 infection, are immunocompromised, or are pregnant. Participants will be monitored for adverse events and serious adverse events by physical examinations, laboratory tests and self-reporting. Blood samples will be collected at serial time points [pre-vaccination/screening (day − 14 to day 0), day 7, day 28, day 180, day 360 post-vaccination] for assessment of antibody and cellular immune parameters. Primary endpoint is the level of anti-SARS-CoV-2 spike immunoglobulins at day 28 post-booster and will be measured against wildtype SARS-CoV-2 and variants of concern. Comprehensive immune profiling of the humoral and cellular immune response to vaccination will be performed.

**Discussion:**

This study will provide necessary data to understand the quantity, quality, and persistence of the immune response to a homologous and heterologous third booster dose of COVID-19 vaccines. This is an important step in developing COVID-19 vaccination programs beyond the primary series.

**Trial registration:**

ClinicalTrials.govNCT05142319. Registered on 2 Dec 2021.

## Administrative information

Note: the numbers in curly brackets in this protocol refer to SPIRIT checklist item numbers. The order of the items has been modified to group similar items (see http://www.equator-network.org/reporting-guidelines/spirit-2013-statement-defining-standard-protocol-items-for-clinical-trials/).

**Table:** 

Title {1}	A randomized, subject-blinded, controlled phase 3 clinical trial to evaluate the safety and immunogenicity of heterologous boost COVID-19 vaccine regimens compared with a homologous boost regimen in healthy individuals [PRIBIVAC]
Trial registration {2a and 2b}.	ClinicalTrials.gov identifier NCT05142319, first published on Dec 2, 2021. https://clinicaltrials.gov/ct2/show/NCT05142319
Protocol version {3}	Version 1 on Dec 2, 2021.
Funding {4}	This study is supported by Singapore National Medical Research Council (NMRC) COVID-19 Research Fund (COVID19RF-0011, COVID19RF-0018).
Author details {5a}	Xuan Ying Poh, I. Russel Lee, Clarissa Lim, Jefanie Teo, Suma Rao, Po Ying Chia, Sean WX Ong, Tau Hong Lee, Ray Lin, David Lye and Barnaby E Young belong to the National Centre for Infectious Diseases (NCID), Singapore. Lisa Ng and Laurent Renia belong to A* STAR Infectious Diseases Labs, Singapore. Ren Ee Chee belong to Singapore Immunology Network, A* STAR, Singapore. Linfa Wang belong to Duke-NUS Medical School, Singapore.
Name and contact information for the trial sponsor {5b}	Trial sponsor: Tan Tock Seng Hospital. Barnaby E Young is the Principal Investigator (PI) of this trial, and any correspondence can be made via this email address: Barnaby_young@ncid.sg
Role of sponsor {5c}	The sponsor will take responsibility for regional conduct, governance and insurance of the trial.

## Introduction

### Background and rationale {6a}

Eradication of the SARS-CoV-2 virus from humans is highly unlikely as the currently available COVID-19 vaccines do not offer long-term “sterilizing” immunity. While the pivotal phase 3 clinical trials of BNT162b2 and mRNA-1273 reported a vaccine efficacy of >95% against symptomatic and severe disease, antibody levels and vaccine effectiveness (VE) declined in the months following vaccinations. For example, 6-month follow-up data for the mRNA-1273 vaccine estimated the half-life for binding antibodies for all participants was 52 days (95% CI 46 to 58), with significantly lower titers with increasing age [1]. Similar long-term data from the BNT162b2 phase 3 clinical trial described a decline in VE from 96.2% (95% CI 93.3 to 98.1) 7 days to < 2 months post-dose 2, to a VE of 90.1% (95% CI 86.6 to 92.9) from 2 months to <4 months, and 83.7% (95% CI 74.7 to 89.9) from 4 months to the data cut-off [2].

Some of the explanations for waning vaccine effectiveness is the emergence of SARS-CoV-2 variants of concern (VOC) which partially evade host immunity from prior infection/vaccination. This includes B.1.617.2 (Delta) and B.1.1.529 (Omicron) [3]. Studies in Singapore and elsewhere have demonstrated that the mRNA vaccines retain high effectiveness against severe infection with Delta or Omicron, higher antibody levels as measured against the ‘wildtype’ spike protein are likely to be necessary to protect against infection [4, 5].

Several studies have demonstrated that a COVID-19 vaccine “booster” dose significantly increases viral neutralization titers against wildtype, Omicron [6–9], and other VOCs. Administration of a third dose of an mRNA vaccine to vaccinated persons at least 6 months after the second dose of an mRNA vaccine led to a 38-fold increase in neutralization titers against Omicron [8]. A third BNT162b2 dose also resulted in 100% of vaccinees displaying neutralization activity against Omicron, as compared to 6% after the full two-dose BNT162b2 vaccination [6]. Vaccine booster combinations tested include homologous mRNA vaccines such as BNT162b2 [6, 7, 9] and mRNA-1273 [7], as well as non-replicating viral vector vaccines AD26.COV2.3 [7] and AZD1222 [9]. However, whether a homologous or heterologous prime-boost-boost vaccine regimen is better in inducing neutralizing antibodies against Omicron, and whether different age groups require different vaccine booster combinations remain unknown. Thus, several fundamental questions about boosters persist: Who needs a booster vaccination? How long after the primary series should it be administered? And, which vaccine should be used?

In Singapore, the mRNA vaccines produced by Pfizer-BioNTech (BNT162b2) and Moderna (mRNA-1273) received interim authorization under the Pandemic Special Access Route (PSAR). Sinovac-CoronaVac has also been added to the National Vaccination Program, primarily for those who are medically ineligible for mRNA vaccines. By 17 January 2022 more than 9 million COVID-19 vaccine doses had been administered, with 88% of Singapore’s resident population fully vaccinated, 90% having received at least one dose, and 53% having received booster shots.

Clinical trials of vaccine candidates which target VOCs are ongoing, though even if successful these vaccines are not expected to be available till late in 2022. Pending the availability of these VOC vaccines, the need for booster vaccinations, particularly in vulnerable populations needs to be assessed. A third dose of the vaccines developed by Pfizer–BioNTech, Moderna, Oxford–AstraZeneca, and Sinovac prompted significant increases in neutralizing antibodies titers when administered several months after the second dose [10].

Preclinical and clinical data have also suggested heterologous prime-boost vaccination may enhance the immune response against SARS-CoV-2 or HIV [11–20]. At day 28 post-boost, SARS-CoV-2 anti-spike IgG concentrations of heterologous schedules (BNT162b2/ChAdOx1 or ChAdOx1/BNT162b2) were higher than that of a homologous ChAdOx1 prime-boost schedule with proven efficacy against COVID-19 disease and hospitalization [15]. In a phase-1 clinical trial conducted in Italy, heterologous prime-boost immunizations with the experimental vaccine GRAd-COV2 and BNT162b2 or ChAdOx1 increased both binding/neutralizing antibodies as well as T cell responses [17]. Due to the large number of potential vaccine combinations, the variation in vaccine types used by programs in different countries, and potentially confounding effects of background infection rates, data relevant to Singapore’s program is not available.

PRIBIVAC will assess a heterologous prime-boost-boost-boost strategy in comparison with a homologous regimen in order to compare short and long-term immunogenicity of different COVID-19 vaccine combinations against the ancestral SARS-CoV-2 as well as different VOCs.

### Objectives {7}

The primary objective is to determine whether heterologous prime-boost-boost COVID-19 vaccine regimens lead to non-inferior humoral immunity compared with homologous prime-boost-boost vaccine regimen against wildtype SARS-CoV-2 and/or 1≥ VOC at day 28 post-vaccination.

The secondary objectives are to (1) determine whether heterologous prime-boost-boost COVID-19 vaccine regimens lead to non-inferior humoral and cellular immunity compared with homologous prime-boost-boost vaccine regimen against wildtype SARS-CoV-2 and/or 1≥ VOC at any other time points (day 1, 7, 180, or 360) post-vaccination, and (2) assess the reactogenicity and safety of heterologous and homologous prime-boost-boost COVID-19 vaccine schedules.

### Trial design {8}

PRIBIVAC is an adaptive, randomized, subject-blinded, controlled, non-inferiority phase 3 trial to assess the immunogenicity and safety of heterologous boost COVID-19 vaccination (intervention groups) compared with a homologous boost regimen (control group). This is an adaptive trial as new intervention groups may also be added depending on what COVID-19 vaccines are authorized for use in Singapore and may include new vaccines or vaccines targeting SARS-CoV-2 variants.

## Methods: participants, interventions, and outcomes

### Study setting {9}

Participants will be enrolled at the National Centre for Infectious Diseases (NCID), Singapore.

### Eligibility criteria {10}

The key eligibility criterion is individuals who had received a homologous primary vaccines series with BNT162b2 or mRNA-1272 at least 6 months prior to study enrolment.

Inclusion criteria:

1. Willing and able to provide informed consent for participation in this study;
2. Aged ≥21years at the time of study enrolment;
3. Received the second dose of BNT162b2 or mRNA-1273 vaccines at least 6 months prior to enrolment;
4. Willing and able to comply with all scheduled visits, vaccination plan, laboratory tests, and other study procedures.

Exclusion criteria:

1. Known history of SARS-CoV-2 or SARS-CoV-1 infection;
2. Previously received an investigational coronavirus vaccine;
3. Previously received a SARS-CoV-2 monoclonal antibody;
4. Current or planned simultaneous participation in another interventional study;
5. A history of anaphylaxis, urticaria, or other significant adverse reaction requiring medical intervention after receipt of a COVID-19 vaccine, or otherwise have a contraindication to one of the available study vaccines per the approved label;
6. Individuals who are immunocompromised (e.g., active leukemia or lymphoma, generalized malignancy, aplastic anemia, solid organ transplant, bone marrow transplant, current radiation therapy congenital immunodeficiency, HIV/AIDS with CD4 lymphocyte count < 200 and patients on immunosuppressant medications);
7. Received systemic immunosuppressants or immune-modifying drugs for >14 days in total within 6 months prior to screening (for corticosteroids >/= 20 mg per day of prednisone equivalent). Topical tacrolimus is allowed if not used within 14 days prior to day 1;
8. Individuals who are pregnant or breastfeeding;
9. Chronic illness that, in the opinion of the study team, is at a stage where it might interfere with trial conduct or completion;
10. Deprived of freedom by an administrative or court order, or in an emergency setting, or hospitalized involuntarily;
11. Current alcohol abuse or drug addiction that might interfere with the ability to comply with trial procedures in the opinion of the study team;
12. Moderate or severe acute illness/infection (according to study team’s judgment) on the day of vaccination, or febrile illness (temperature ≥ 37.5°C). A prospective participant should not be included in the study until the condition has resolved or the febrile event has subsided.

### Who will take informed consent? {26a}

The principal investigator (PI) or medically qualified co-investigators are responsible for ensuring freely given consent is obtained from each potential participant prior to the conduct of any protocol-specific procedures.

### Additional consent provisions for collection and use of participant data and biological specimens {26b}

Leftover biological specimen and/or data may be used for future research, subject to participant consent.

## Interventions

### Explanation for the choice of comparators {6b}

The PRIBIVAC study aims to assess the immunogenicity and safety of heterologous boost COVID-19 vaccine regimens compared with a homologous boost regimen.

Circulating antibody levels wane following vaccination and COVID-19 infection. In our previous study of COVID-19 recovered patients, ~60% of the cohort (*n* = 164) retained >30% inhibition level of neutralizing antibodies against SARS-CoV-2 at 6 months post infection [5]. Data from individuals vaccinated with mRNA-1273 showed gradually declining neutralizing antibody titers by 6 months post inoculation [1].

A third dose of the vaccines developed by Pfizer–BioNTech, Moderna, Oxford–AstraZeneca, and Sinovac elicited significant increases in neutralizing antibodies titers when administered several months after the second dose [10]. However, there is limited data as to whether a homologous booster regimen can enhance protection against emerging VOC. Due to the large number of potential vaccine combinations, the variation in vaccine types used by programs in different countries, and potentially confounding effects of background infection rates, data relevant to Singapore’s program is not available.

COVAXIN® (Bharat Biotech) was approved by WHO under the EUL procedure on 3 Nov 2021 for use in two doses for primary vaccination and is available in Singapore via the Special Access Route (SAR). A randomized, double-blind, placebo-controlled, multicenter, phase 3 clinical trial in India has confirmed the safety and efficacy of COVAXIN® against laboratory-confirmed symptomatic COVID-19 disease in adults [21]. However, the efficacy of COVAXIN® against emerging VOCs is unknown and is not currently being studied in booster trials.

### Intervention description {11a}

The booster vaccine for the control arm will be a homologous mRNA vaccine (e.g., BNT162b2 + BNT162b2 + BNT162b2 or mRNA-1273 + mRNA-1273 + mRNA-1273), while for individuals randomized to intervention group 1 the mRNA booster vaccine administered will be heterologous to the primary series (e.g., BNT162b2 + BNT162b2 + mRNA-1273 or mRNA-1273 + mRNA-1273 + BNT162b2). The booster vaccine candidates for intervention groups 2–4 will be an alternative non-mRNA COVID-19 vaccine. At the time of writing (25 Jan, 2022), vaccine candidates for intervention groups 3 and 4 have not been confirmed.

- Control group: Homologous mRNA booster vaccine
- Intervention group 1: Heterologous mRNA booster vaccine
- Intervention group 2: COVAXIN®
- Intervention group 3: Non-mRNA booster vaccine B
- Intervention group 4: Non-mRNA booster vaccine C

### Criteria for discontinuing or modifying allocated interventions {11b}

The participant may be withdrawn prematurely from the trial if he/she withdraws consent to participate in the study. In such instances, the withdrawal will be applicable to both data collection as well as vaccination.

Participants may voluntarily decline the booster vaccination, or they may be withdrawn by their attending physician and/or by the study investigator(s) on the basis of the participant’s best interest. Even if the participant did not receive the booster vaccination, collected samples will still be analyzed up until the point the participant withdraws consent.

All participants, including those who declined the booster vaccination, will be followed up until 12 months, unless the subject withdraws informed consent or is lost to follow-up or has died.

Safety and futility will be reviewed by an independent Data and Safety Monitoring Board (DSMB) after the first 10 participants in each of the intervention arms have completed assessments at study day 28. DSMB may recommend discontinuation of a study arm on the basis of safety concerns or futility in immunogenicity.

### Strategies to improve adherence to interventions {11c}

Booster dose will be given once only; patient adherence is not applicable.

### Relevant concomitant care permitted or prohibited during the trial {11d}

There will be no restrictions on diet, exercise, or concomitant medication imposed on participants, except for the restriction of receiving any other COVID-19 vaccines outside of this study.

### Provisions for post-trial care {30}

Unless there are any serious adverse events that need to be followed up closely, no post study follow-up and procedures need to be performed after the final study visit. If the participant follows the directions of the study team and is physically injured due to the procedure given under the plan for this study, NCID/TTSH will pay the medical expenses for the treatment of that injury.

### Outcomes {12}

The outcomes are shown in Table 1.

**Table 1:** Summary of objectives and outcome measures

Objectives	Outcome measures	Timepoint(s)
**Primary objective**		
To determine whether heterologous prime-boost-boost COVID-19 vaccine regimens lead to non-inferior humoral immunity compared with homologous prime-boost-boost vaccine regimen against wildtype SARS-CoV-2 and/or 1≥ VOC	Level of SARS-CoV-2 anti-spike immunoglobulins	Day 28
**Secondary objectives**		
To determine whether heterologous prime-boost-boost COVID-19 vaccine regimens lead to non-inferior humoral and cellular immunity compared with homologous prime-boost-boost vaccine regimen against wildtype SARS-CoV-2 and/or 1≥ VOC	Level of SARS-CoV-2 anti-spike immunoglobulins Level of SARS-CoV-2 neutralizing antibodies Quantitative T cell responses to spike proteins	Days 1, 7, 180, 360 Days 1, 7, 28, 180, 360 Days 1, 7, 28, 180, 360
To assess the reactogenicity and safety of heterologous and homologous prime-boost-boost COVID-19 vaccine schedules	Solicited local and systemic reaction Changes from baseline in laboratory safety measures Unsolicited adverse events (AEs) Serious adverse events (SAEs), AEs of special interest (e.g., myocarditis, pericarditis), medically attended AEs	Day 7 Day 7 Day 28 Throughout the study
**Exploratory objectives**		
To determine whether vaccine efficacy differs between heterologous and homologous prime-boost-boost regimens	PCR-confirmed COVID-19 infections as recognized by the Ministry of Health, Singapore	Throughout the study
To determine whether the administered mRNA vaccine (control group and intervention group 1 only) can be detected in the blood at ~1 week post-vaccination	qRT-PCR using primers targeting the sequence of the mRNA for SARS-CoV-2 spike protein	Day 7

### Participant timeline {13}

The participant timeline is shown in Table 2.

**Table 2:**
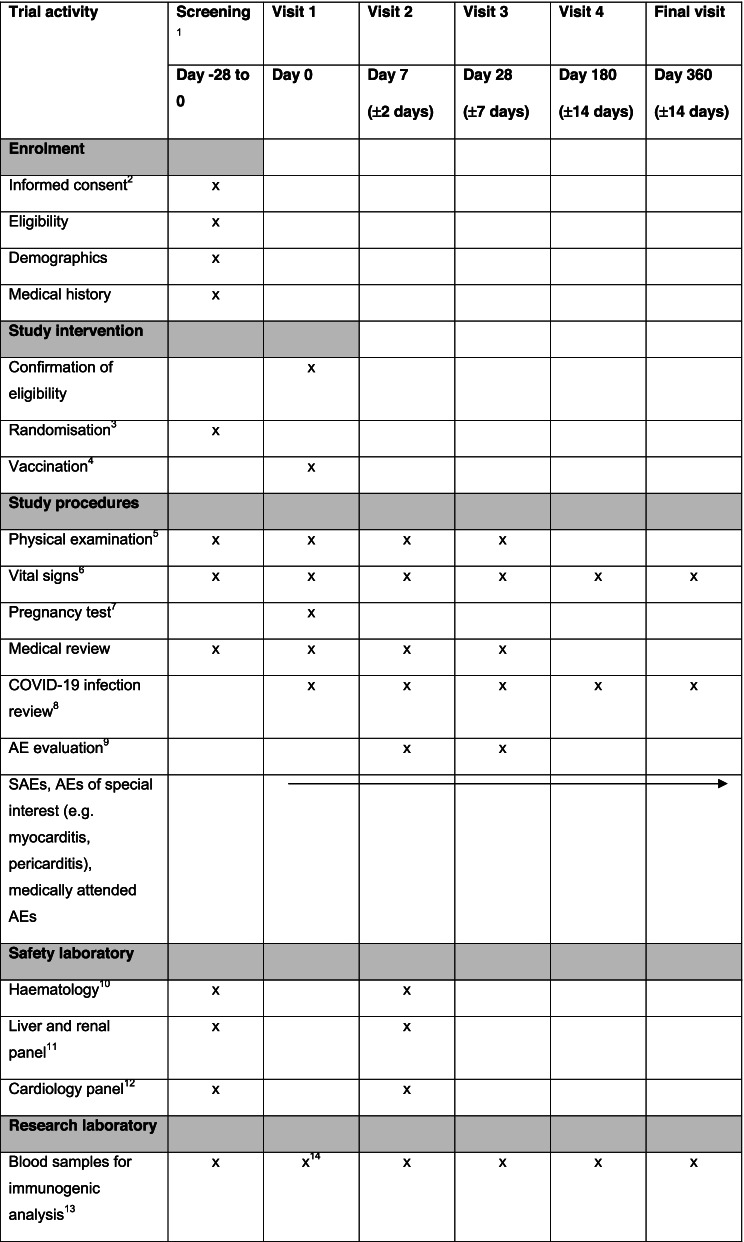
Trial schedule

^1^Screening visit may be performed on the same day as visit 1

^2^Informed consent must be signed prior to initiating any study procedures;

^3^Randomization is subjected to vaccine availability;

^4^Visit 1: third vaccine shot for participant (only one vaccine booster dose will be administered for the study);

^5^Physical examination will be done by a qualified study team member or a treating licensed healthcare provider. The physical examination on visit 1 will be conducted during screening prior to vaccination;

^6^Vital signs include pulse, systolic and diastolic blood pressure, respiratory rate, and body temperature prior to vaccination and blood collection;

^7^For women of child bearing potential, a urine pregnancy test will be conducted;

^8^At each study visit, a review for PCR-confirmed COVID-19 infection will be conducted. In most cases, such information will be available in the participant’s HealthHub mobile app;

^9^All AEs including a list of solicited and other events will be recorded for 7 days after vaccination. Unsolicited AEs will be recorded up to day 28 after vaccination. SAE will be recorded from study’s vaccination until the end of study period at 12 months. They will be assessed via the listed study procedures, safety laboratory tests, and participant self-recorded diary;

^10^Hematology tests include full blood count with differential and platelet counts;

^11^Liver panel includes albumin, total bilirubin, alkaline phosphatase (ALP), and alanine transaminase (ALT); Renal panel includes sodium, potassium, and creatinine;

^12^Cardiology panel includes creatine kinase and troponin;

^13^Blood samples will be collected at NCID research clinic, de-identified/coded before dispatch to research laboratories such as A*STAR Singapore Immunology Network, Duke-NUS and NCID’s National Public Health Laboratory. The study team based at NCID research clinic will maintain the codes linking the blood samples to its donor. Subjected to participant consent, any de-identified leftover blood samples may also be analyzed for exploratory research to find new scientific information about coronaviruses and related diseases, which may occur locally or overseas;

^14^Blood sample for immunogenic studies may be taken at Visit 1 instead of screening visit if blood draw at screening is not possible. The study team will make every effort to complete all blood-taking in one seating to minimize the number of needle pricks.

### Sample size {14}

Based on data from our ongoing COVID-19 vaccine immune-monitoring study (SCOPE) [22], the mean level of SARS-CoV-2 anti-spike immunoglobulins was 84% (SD=15%) at 28 days after the second dose. This was determined using a SARS-CoV-2 surrogate virus neutralization test (sVNT) that detects total immuno-dominant neutralizing antibodies targeting the viral spike (S) protein receptor-binding domain in an isotype- and species-independent manner. We expect immunogenicity will be boosted back to the same level after the third booster dose in the control arm. Assuming an immunogenicity level of 81% in an intervention arm and a non-inferiority margin of − 10%, a sample size of 87 subjects per arm is needed to conclude non-inferiority of the intervention arm against the control arm with 80% power. The sample size is calculated at a one-sided 2.5% significance level and accounts for an attrition rate of 15%. A sample size of 100 subjects per arm will provide a power of 85.1% based on the above assumptions and calculations.

Since recruitment of subjects to intervention arms may be activated in a staggered way depending on availability of the vaccines, and recruitment to the control arm will continue until the target sample size of 100 is achieved in each intervention arm, we prepare to enroll a double sample size in the control arm for the purpose of study planning. This gives a total sample size of up to 600 subjects.

### Recruitment {15}

Recruitment will occur over 6–9 months with 1-year follow-up period.

## Assignment of interventions: allocation

### Sequence generation {16a}

Randomization will be performed using randomized permuted blocks and stratified by the following criteria:

1. Age (<60 years, ≥60 years)
2. Time from 2nd vaccine dose administered (6–9 months, >9 months)
3. Primary vaccine series (BNT162b2 or mRNA-1273)

Eligible participants will be randomized in equal proportions to each of the study arms that are open for randomization. Enrolment to a study arm will be discontinued if recommended by DSMB or if the target sample size of 100 in the study arm has been reached. In the scenario where recruitment is discontinued in any of the intervention arms from 1 to 4 at the interim analysis, randomization to the control arm may continue beyond 100 participants until the target sample size of 100 participants has been reached in the remaining intervention arms.

### Concealment mechanism {16b}

Randomization will be performed using a web-based randomization system hosted by Singapore Clinical Research Institute (SCRI). Password secured accounts will be assigned to the site personnel responsible for the randomization, where he/she can log into the randomization system using the Internet. Allocation concealment will be maintained until the registration and randomization process has been completed.

### Implementation {16c}

The study team from SCRN in charge of participant enrolment will perform the randomization using the web-based randomization system.

## Assignment of interventions: blinding

### Who will be blinded {17a}

Vaccine allocation will be single-blind, i.e. only participants will be blinded. This is to reduce the risk of bias in participant-reported adverse events. Data analysts are not blinded.

### Procedure for unblinding if needed {17b}

Blinding will be maintained for the participants until their day 28 visit (visit 3).

## Data collection and management

### Plans for assessment and collection of outcomes {18a}

All physical examinations will be performed by the investigator or co-investigator. It includes cardiovascular, respiratory, abdomen, neurological, musculoskeletal, and skin assessments. Vital signs including pulse rate, systolic and diastolic blood pressure, respiratory rate, and body temperature will also be recorded.

The participant will be given a diary to record all the local and general symptoms experienced after receiving the vaccination. Local symptoms may include pain, redness, swelling, bruising, itchiness, muscle ache or movement limitation at the injection site. General symptoms may include headache, fever, nausea, vomiting, sore throat, cough, runny nose, tiredness, and red (sore) eyes, as well as symptoms suggestive of COVID-19 infections (e.g., fever, cough, and shortness of breath). This diary will cover 7 days post-vaccination and must be returned to the study team when the participant comes for the next visit.

Safety will be assessed via physical examinations, vital sign measurements, medical reviews, AE and SAE evaluation, safety laboratory tests, and participant self-recorded diary. Blood samples will be taken during screening or day 0 and at the follow-up visits at days 7, 28, 180, and 360 post-enrollment for evaluation of immunogenicity and/or safety. All adverse events will be entered in the appropriate eCRF (including seriousness, grade, severity, relationship to the intervention procedure, and action taken) and in the source documents.

### Plans to promote participant retention and complete follow-up {18b}

The study team will remind participants prior to their follow-up visits to minimize any missed appointments. Participants will be reimbursed for their time, inconvenience, and transportation costs for every study visit they complete.

### Data management {19}

Paper Case Report Forms (CRFs) will be used for initial data collection. These will be transcribed to an eCRF using Research Electronic Data Capture (REDCap). The trial database will include information on demographics, medical history, vaccine arm allocation, vital signs, laboratory investigation tests, as well as AEs and SAEs. Paper documents will be maintained and stored in a locked office, with access restricted to study personnel. All electronic documents with participant information will be password-protected.

Study monitors will visit the study site periodically to assess data quality and study integrity. Study monitors will review the study records on site to directly compare them with source documents, discuss the conduct of the study with the Investigator, and verify that the facilities and workflow are compliant with Good Clinical Practice (GCP). Such visits will be scheduled in advance to allow for logistical arrangements to be made. In addition, the study may be evaluated by government inspectors who must be allowed access to CRFs, source documents, and other study files.

### Confidentiality {27}

All study findings and documents will be regarded as confidential. The investigators and other study personnel must not disclose such information without prior written approval from the PI. Participant confidentiality will be strictly maintained to the extent possible under the law and local hospital policy. Identifiable information will be removed from any published data.

### Plans for collection, laboratory evaluation, and storage of biological specimens for genetic or molecular analysis in this trial/future use {33}

Blood samples will be collected at NCID research clinic, de-identified/coded before dispatch to collaborating research laboratories such as A*STAR ID Labs and Singapore Immunology Network, Duke-NUS, and NCID’s NPHL for immunogenic studies as described below:

#### Antibody response assays

To determine the presence and levels of anti-SARS-COV-2 in human sera using S-protein flow cytometry-based (SFB) assay. Cells expressing the full S protein in its native configuration will be seeded at 1.5 × 10^5^ cells per well in 96 well V-bottom plates. The cells will be first incubated with human serum (diluted 1:100 in 10% FBS) before a secondary incubation with a double stain, consisting of Alexa Fluor 647-conjugated anti-human IgG (diluted 1:500) and propidium iodide (PI; diluted 1:2500). Cells were read on BD Biosciences LSR4 laser and analyzed using FlowJo (Tree Star). A standard ELISA will also be used with the whole spike-protein, RBD fragment, or peptides encompassing dominant epitopes of the S and N proteins immobilized in the microplate wells. To identify the repertoire of epitopes induced by the vaccines, a peptide library ELISA will be used. This will provide fine resolution of the antibody responses against the S-proteins at different time points and help to determine whether there is a fixation of the antibody response to certain epitopes (antigenic sin).

To examine the neutralizing capacity of the antibodies in the human sera, two different assays will be performed: the sVNT and the pseudovirus or live-virus assay inhibition.

#### T cell response assays

Quantitative T-cell responses to the vaccines will be measured using SARS-CoV-2 peptides from spike protein to stimulate the PBMCs isolated from the donor’s blood by ELISPOT or flow cytometry. To detect CD8 T-cells, PBMCs will be stimulated with a peptide pool consisting of peptides 8–10 amino acids in length. Negative controls using media and DMSO (~0.5%) will be used as reference. Assays will be performed in duplicates. The cell and peptides will be incubated in ELISpot 96-well plates. After 24 h, the plates will be washed and assayed for IFNγ. To detect CD4 Th1/2 cells, PBMCs will be stimulated with a peptide pool consisting of peptides that are 15 amino acids in length. After 24 h, the plates will be washed and assayed for IFNγ (Th1) or IL4/IL5/IL13 (Th2). Spot forming units (SFU) will be measured using IRIS reader (MabTech). SARS-CoV-2-specific T effector subsets will be tested after stimulation with pooled SARS-CoV-2 PepTivator® S, S1, peptides (0.6 nmol/mL each) (Miltenyi Biotec) for 6 h. Brefeldin A and Monesin (ThermoFisher Scientific) was added at 2 h post-stimulation. Cells were stained with surface stain markers in the dark at room temperature for 20 min, followed by fixation and permeabilization for 20 min with Foxp3/Transcription Factor Staining Buffer Set (ThermoFisher Scientific). Permeabilized cells were then stained for intracellular cytokines for 20 min. Cells were then acquired with the Cytek^TM^ Aurora cytometer running SpectroFlo® Version 2.2.0.3 with automated unmixing. Compensation and analysis of flow cytometry data was performed with FlowJo Version 10.6.1.

The study team based at the NCID research clinic will maintain the codes linking the blood samples to its donor. Subjected to participant consent, any de-identified leftover blood samples may also be analyzed for exploratory research to find new scientific information about coronaviruses and related diseases, which may occur locally or overseas.

## Statistical methods

### Statistical methods for primary and secondary outcomes {20a}

#### Safety analyses

Data of AEs, SAEs, grade 3 and 4 clinical or laboratory AEs will be summarized by the study arm with frequency and proportion of participants having the event, as well as the number of events. For SAEs and grade 3 and 4 AEs, the proportion of participants with the events will be provided together with its 95% confidence interval (CI).

#### Efficacy analyses

Graphical plots will be produced for the various immunity endpoints to depict the change of immunity levels over time. Levels of SAS-CoV-2 anti-spike immunoglobulins will be summarized by study arm and by randomization stratification factors. Mean difference and its 95% confidence interval in levels of SAS-CoV-2 anti-spike immunoglobulins between an intervention arm and the control arm will be estimated from a general linear regression model which adjusts for level of SAS-CoV-2 anti-spike immunoglobulins at baseline, and randomization stratification factors. If the lower bound of the 95% CI falls above − 10% (the pre-specified non-inferiority margin), non-inferiority of the intervention arm will be concluded. Analysis of repeated measurements of immunity endpoints will be performed with the use of mixed effects models that adjust for baseline values and randomization stratification factors. Log-transformation of the data may be applied as needed before the analyses.

### Interim analyses {21b}

Interim analyses will be performed for DSMB review after 10 participants from each of the intervention arms from 1 to 4 have completed assessments at study day 28. DSMB may recommend discontinuation of participant enrolment to a study arm if any of the following criteria is met:

- Proportion of participants with SAE is at least 25% greater (in absolute difference) in the intervention arm compared with the control arm
- Proportion of participants with Grade 3 and 4 AEs is at least 25% greater (in absolute difference) in the intervention arm compared with the control arm
- Geometric mean ratio of anti-SARS-CoV-2 between the intervention arm and the control arm falls below 0.60.

The above guidelines may be revised in the DSMB charter, which implies the stopping guidelines will take precedence should there be any difference in the guidelines between the protocol and the DSMB charter.

### Methods for additional analyses (e.g., subgroup analyses) {20b}

Analysis will be stratified by age (<60 years, ≥60 years), time from 2nd vaccine dose administered (6-9 months, >9 months), and primary vaccine series (BNT162b2 or mRNA-1273).

### Methods in analysis to handle protocol non-adherence and any statistical methods to handle missing data {20c}

Even if the participant did not receive the booster vaccination, collected samples will still be analyzed up until the point the participant withdraws consent. Participants who drop out of the study will not be replaced.

### Plans to give access to the full protocol, participant-level data, and statistical code {31c}

Datasets analyzed during the study will be available from the corresponding author on reasonable request.

## Oversight and monitoring

### Composition of the coordinating center and trial steering committee {5d}

This study is led by NCID’s Singapore Infectious Disease Clinical Research Network (SCRN) and supported by Singapore Clinical Research Institute (SCRI). SCRN is responsible for project management, subject recruitment and data entry. SCRI is responsible for project and data management, as well as regulatory compliance/ study monitoring.

### Composition of the data monitoring committee, its role, and reporting structure {21a}

An independent DSMB has been established to monitor the study. The DSMB will evaluate the study design and protocol, and review the accumulative data of safety and immunogenicity, as well as subject enrolment, protocol deviations, and data quality. The DSMB will meet after 10 participants in each intervention group have completed assessments at study day 28. Since participant recruitment may start at different time points for intervention groups 1 to 4 depending on vaccine availability and local regulatory approval, separate DSMB meetings may be needed for data review of intervention groups 1 to 4.

A DSMB Charter will be developed describing the scope of data to be reviewed, memberships of DSMB, terms of reference, decision-making process, and timing and frequency of interim analyses (with specification of stopping guidelines). An interim analysis report will be prepared for each DSMB data review, which will include a summary data on participant enrolment, data quality, demographic and baseline characteristics, protocol deviation, safety data, and immunogenicity data. Following a review of interim data, DSMB may recommend discontinuation of participant enrolment to a study arm on the basis of safety concern and/or futility in immunogenicity compared with the control arm. The DSMB may request additional data review if necessary.

### Adverse event reporting and harms {22}

Advent event reporting and harms are shown in Table 3.

**Table 3:** Adverse event reporting and harms

**Adverse events (AEs)**	
Safety will be assessed using the FDA Guidance Document (2007): Toxicity Grading Scale for Healthy Adult and Adolescent Volunteers Enrolled in Preventive Vaccine Clinical Trial (http://www.fda.gov/BiologicsBloodVaccines/GuidanceComplianceRegulatoryInformation/Guidances/Vaccines/ucm074786.htm). Participants will be monitored up to Visit 2 (Day 7) for the occurrence and nature of any AEs. All AEs will be entered in the appropriate eCRF (including seriousness, grade, severity, relationship to the IP, and action taken) and in the source documents. The hospital laboratory will perform investigational tests as specified in the trial schedule, including full blood count (with differential blood count and platelet count), liver panel (total bilirubin, ALP, ALT), renal panel (sodium, potassium, and creatinine), creatine kinase, troponin, and pregnancy test if indicated. For out-of-range values, clinical laboratory reports must be reviewed by a physician within 24 h of receipt. Out-of-range values will be evaluated as either clinically significant (CS) or not clinically significant (NS). By definition, a value flagged as “CS” must be entered on the AE page in the CRF. The test may be repeated at the Investigator’s discretion. The Investigator may use his own judgment to determine whether the abnormal finding has sufficient reasons to immediately withdraw the participant from the study.	
**Collecting, recording, and reporting of “Unanticipated Problems Involving Risk to Subjects or Others” (UPIRTSO) events to the National Healthcare Group (NHG) Domain Specific Review Boards (DSRB)**	
UPIRTSO events refers to problems, in general, to include any incident, experience, or outcome (including AEs) that meets all of the following criteria: 1. Unexpected, in terms of nature, severity, or frequency of the problem as described in the study documentation (eg: Protocol, Consent documents, etc). 2. Related or possibly related to participation in the research. Possibly related means there is a reasonable possibility that the problem may have been caused by the procedures involved in the research; and 3. Risk of harm. This suggests that the research places participants or others at a greater risk of harm (including physical, psychological, economic, or social harm) than was previously known or recognized. For urgent reporting, all problems involving local deaths, whether related or not, should be reported immediately – within 24 h after first knowledge by the NHG investigator. For expedited reporting, all other problems must be reported as soon as possible but not later than 7 calendar days after first knowledge by the NHG investigator.	
**Collecting, recording, and reporting of serious adverse events (SAEs) to the Health Science Authority (HSA)**	
A SAE is defined as any untoward medical occurrence that: results in death, is life-threatening (immediate risk of death), requires inpatient hospitalization or prolongation of existing hospitalization, results in persistent or significant disability/incapacity, results in congenital anomaly/birth defect, or is a medically important event. Medical and scientific judgment should be exercised in determining whether an event is an important medical event. An important medical event may not be immediately life-threatening and/or result in death or hospitalization. However, if it is determined that the event may jeopardize the subject and/or may require intervention to prevent one of the other AE outcomes, the important medical event should be reported as serious. All SAEs that are unexpected and related to the study drug will be reported. The investigator is responsible for informing HSA no later than 15 calendar days after first knowledge that the case qualifies for expedited reporting. Follow-information will be actively sought and submitted as it becomes available. For fatal or life-threatening cases, HSA will be notified as soon as possible but no later than 7 calendar days after first knowledge that a case qualifies, followed by a complete report within 8 additional calendar days.	

### Frequency and plans for auditing trial conduct {23}

Clinical site monitoring is conducted to ensure that the rights and well-being of trial subjects are protected and that the reported trial data are accurate, complete, and verifiable. Clinical monitoring also ensures the conduct of the trial is in compliance with the currently approved protocol/ amendment(s), ICH, GCP, and with applicable regulatory requirement(s) and sponsor requirement(s). Clinical monitoring will also verify that any critical study procedures are completed following specific instructions in the protocol-specific manual of procedures. Details of clinical site monitoring are documented in a clinical monitoring plan (CMP). The CMP describes in detail who will conduct the monitoring, at what frequency monitoring will be done, at what level of detail monitoring will be performed, and the distribution of monitoring reports. Monitoring visits will include, but are not limited to, review of regulatory files, accountability records, CRFs, ICFs, medical and laboratory reports, site study intervention storage records, training records, and protocol and GCP compliance.

### Plans for communicating important protocol amendments to relevant parties (e.g., trial participants, ethical committees) {25}

During the trial, any amendments to the protocol or consent materials will be approved by the Ethics Committee (NHG DSRB) before implementation. Participants will be informed in a timely manner of any new information that becomes available during the course of the study that may affect their willingness to continue study participation.

### Dissemination plans {31a}

Following completion of the study, results will be published in a medical/scientific journal. Preliminary results may be released by the sponsor to help inform policy-making decisions pertaining to COVID-19 vaccination booster regimens.

## Discussion

PRIBIVAC is an adaptive, randomized, subject-blinded, controlled trial to assess the immunogenicity and safety of heterologous boost COVID-19 vaccination (intervention groups) compared with a homologous boost regimen (control group). The booster vaccine for the control arm will be the homologous mRNA vaccine (e.g., BNT162b2 + BNT162b2 + BNT162b2 or mRNA-1273 + mRNA-1273 + mRNA-1273), while for individuals randomized to intervention group 1 the mRNA booster vaccine administered will be heterologous to the primary series (e.g., BNT162b2 + BNT162b2 + mRNA-1273 or mRNA-1273 + mRNA-1273 + BNT162b2). The booster vaccine candidates for intervention groups 2–4 will be an alternative COVID-19 vaccine. New intervention groups may also be added depending on what COVID-19 vaccines are authorized for use in Singapore, and may include new vaccines or vaccines targeting SARS-CoV-2 variants.

Since access to non-mRNA vaccine candidates is dependent on various factors outside the control of the study team availability of all vaccines at the outset of the study may be limited. Participants will be randomized at equal probability to the available intervention groups at the time of randomization. This will reduce the risk of bias (e.g., participant preference for a certain group) compared with a non-randomized design. While the ideal scenario is to have all the selected vaccines approved prior to the start of recruitment, due to the unknown time of availability of vaccine candidates A (i.e., COVAXIN®), B and C, and the urgency of the current situation (i.e., emerging VOC), the study was initiated with the control and intervention group 1.

With the emergence of increasingly infectious VOCs such as the Omicron variant and the increasing frequency of vaccine breakthrough infections, an effective long-term COVID-19 immunization strategy is urgently needed. Currently, there are no COVID-19 vaccine booster trials to evaluate the efficacy of newly approved vaccines such as COVAXIN® against the primary mRNA series (BNT162b2 and mRNA-1273) as a booster dose to induce protective immunity against emerging VOCs. In addition, the effects of aging on the immune system result in elderly individuals not responding to immune challenges (i.e., vaccination) as robustly as the young [23], thus long-term COVID-19 immunization strategies should take into account different age groups within the population. Immunosenescence, which is the gradual deterioration of the immune system with age, starts to affect people’s health at about 60 [24]. However, clinical trials that investigate the immunogenicity of COVID-19 vaccination regimens in the older (≥60 years old) versus the younger (<60 years old) age groups are currently lacking. PRIBIVAC will offer insights into the efficacy, in terms of protective immunity against the ancestral SARS-CoV-2 as well as emerging VOCs, of different COVID-19 booster vaccine combinations in different age groups. This trial can help inform the choice of vaccine booster for targeted age groups in the upcoming COVID-19 booster vaccine regimens. In addition, the long-term follow-up (up to 1 year post-vaccination) to monitor the persistence of humoral and cellular immunity in vaccinees can help inform other countries with regards to the appropriate length of time after the primary COVID-19 vaccine series to administer the 3^rd^ or 4^th^ booster dose.

## Trial status

The trial began recruiting participants in Oct 2021. Recruitment is expected to conclude by mid-2022, with the final participant visit by mid-2023.

## Abbreviations

AEs: Adverse events
CMP: Clinical monitoring plan
CRF: Case report form
DSMB: Data and Safety Monitoring Board
DSRB: Domain Specific Review Boards
eCRF: Electronic case report form
EUL: Emergency Use List
GCP: Good clinical practice
HSA: Health Sciences Authority
ICF: Informed consent form
mRNA: Messenger ribonucleic acid
NCID: National Centre for Infectious Diseases
NHG: National Healthcare Group
NPHL: National Public Health Laboratory
PSAR: Pandemic Special Access Route
PCR: Polymerase chain reaction
PI: Principal investigator
qRT-PCR: Quantitative reverse transcription-polymerase chain reaction
REDCap: Research Electronic Data Capture
SAEs: Serious adverse events
SAR: Special Access Route
SCRI: Singapore Clinical Research Institute
SCRN: Singapore Clinical Research Network
sVNT: Surrogate virus neutralization test
TTSH: Tan Tock Seng Hospital
UPIRTSO: Unanticipated Problems Involving Risk to Subjects or Others
VE: Vaccine efficacy
VOCs: Variants of concern
WHO: World Health Organization

## References

[1] Doria-Rose N, Suthar MS, Makowski M, O'Connell S, McDermott AB, Flach B (2021). Antibody persistence through 6 months after the second dose of mRNA-1273 vaccine for COVID-19. N Engl J Med.

[2] Thomas SJ, Moreira ED, Kitchin N, Absalon J, Gurtman A, Lockhart S (2021). Safety and efficacy of the BNT162b2 mRNA COVID-19 vaccine through 6 months. N Engl J Med.

[3] CDC. Science Brief: Emerging SARS-CoV-2 Variants. https://www.cdc.gov/coronavirus/2019-ncov/science/science-briefs/scientific-brief-emerging-variants.html#. Accessed 04 Mar 2022.

[4] Planas D, Veyer D, Baidaliuk A, Staropoli I, Guivel-Benhassine F, Rajah MM (2021). Reduced sensitivity of SARS-CoV-2 variant Delta to antibody neutralization. Nature..

[5] Chia WN, Zhu F, Ong SWX, Young BE, Fong SW, Le Bert N (2021). Dynamics of SARS-CoV-2 neutralising antibody responses and duration of immunity: a longitudinal study. Lancet Microbe.

[6] Planas D, Saunders N, Maes P, Guivel-Benhassine F, Planchais C, Buchrieser J, et al. Considerable escape of SARS-CoV-2 omicron to antibody neutralization. Nature. 2022;602(7898):671–5. 10.1038/s41586-021-04389-z 35016199

[7] Garcia-Beltran WF, St Denis KJ, Hoelzemer A, Lam EC, Nitido AD, Sheehan ML, et al. mRNA-based COVID-19 vaccine boosters induce neutralizing immunity against SARS-CoV-2 omicron variant. Cell. 2022;185(3):457–66 e4. 10.1016/j.cell.2021.12.033 PMC8733787 34995482

[8] Schmidt F, Muecksch F, Weisblum Y, Da Silva J, Bednarski E, Cho A, et al. Plasma neutralization of the SARS-CoV-2 omicron variant. N Engl J Med. 2022;386(6):599–601. 10.1056/NEJMc2119641 PMC8757565 35030645

[9] Dejnirattisai W, Huo J, Zhou D, Zahradnik J, Supasa P, Liu C (2022). SARS-CoV-2 omicron-B.1.1.529 leads to widespread escape from neutralizing antibody responses. Cell..

[10] Callaway E (2021). COVID vaccine boosters: the most important questions. Nature..

[11] Tenbusch M, Schumacher S, Vogel E, Priller A, Held J, Steininger P (2021). Heterologous prime-boost vaccination with ChAdOx1 nCoV-19 and BNT162b2. Lancet Infect Dis.

[12] Lu S (2009). Heterologous prime-boost vaccination. Curr Opin Immunol.

[13] He Q, Mao Q, An C, Zhang J, Gao F, Bian L (2021). Heterologous prime-boost: breaking the protective immune response bottleneck of COVID-19 vaccine candidates. Emerg Microbes Infect.

[14] Liu J, Xu K, Xing M, Zhuo Y, Guo J, Du M (2021). Heterologous prime-boost immunizations with chimpanzee adenoviral vectors elicit potent and protective immunity against SARS-CoV-2 infection. Cell Discov.

[15] Liu X, Shaw RH, Stuart ASV, Greenland M, Aley PK, Andrews NJ (2021). Safety and immunogenicity of heterologous versus homologous prime-boost schedules with an adenoviral vectored and mRNA COVID-19 vaccine (com-COV): a single-blind, randomised, non-inferiority trial. Lancet..

[16] Li W, Li X, Zhao D, Liu J, Wang L, Li M (2021). Heterologous prime-boost with AdC68- and mRNA-based COVID-19 vaccines elicit potent immune responses in mice. Signal Transduct Target Ther.

[17] Agrati C, Capone S, Castilletti C, Cimini E, Matusali G, Meschi S (2021). Strong immunogenicity of heterologous prime-boost immunizations with the experimental vaccine GRAd-COV2 and BNT162b2 or ChAdOx1-nCOV19. NPJ Vaccines.

[18] Brown SA, Surman SL, Sealy R, Jones BG, Slobod KS, Branum K (2010). Heterologous prime-boost HIV-1 vaccination regimens in pre-clinical and clinical trials. Viruses..

[19] Mogus AT, Liu L, Jia M, Ajayi DT, Xu K, Kong R, et al. Virus-like particle based vaccines elicit neutralizing antibodies against the HIV-1 fusion peptide. Vaccines (Basel). 2020;8(4):765. 10.3390/vaccines8040765 PMC7765226 33333740

[20] Palgen JL, Feraoun Y, Dzangue-Tchoupou G, Joly C, Martinon F, Le Grand R (2021). Optimize prime/boost vaccine strategies: trained immunity as a new player in the game. Front Immunol.

[21] Ella R, Reddy S, Blackwelder W, Potdar V, Yadav P, Sarangi V (2021). Efficacy, safety, and lot-to-lot immunogenicity of an inactivated SARS-CoV-2 vaccine (BBV152): interim results of a randomised, double-blind, controlled, phase 3 trial. Lancet..

[22] Renia L. Durable T cell responses contrast with faster antibody waning in BNT162b2-vaccinated elderly at 6 month. 10.21203/rs.3.rs-1103804/v1. In press 09 Dec 2021. https://www.researchsquare.com/article/rs-1103804/v1

[23] Montecino-Rodriguez E, Berent-Maoz B, Dorshkind K (2013). Causes, consequences, and reversal of immune system aging. J Clin Invest.

[24] Lawton G (2020). You're only as young as your immune system. New Sci.

